# Meta analysis of whole-genome linkage scans with data uncertainty: an application to Parkinson's disease

**DOI:** 10.1186/1471-2156-8-44

**Published:** 2007-07-02

**Authors:** Albert Rosenberger, Manu Sharma, Bertram Müller-Myhsok, Thomas Gasser, Heike Bickeböller

**Affiliations:** 1 Georg-August-University Göttingen, Medical School, Department of Genetic Epidemiology, Germany; 2 Eberhard-Karl-University Tübingen, Centre of Neurology, Hertie Institute for Clinical Brain Research, Germany; 3 Max-Planck Institute for Psychiatry, Munich, Germany

## Abstract

**Background:**

Genome wide linkage scans have often been successful in the identification of genetic regions containing susceptibility genes for a disease. Meta analysis is used to synthesize information and can even deliver evidence for findings missed by original studies. If researchers are not contributing their data, extracting valid information from publications is technically challenging, but worth the effort. We propose an approach to include data extracted from published figures of genome wide linkage scans. The validity of the extraction was examined on the basis of those 25 markers, for which sufficient information was reported. Monte Carlo simulations were used to take into account the uncertainty in marker position and in linkage test statistic. For the final meta analysis we compared the Genome Search Meta Analysis method (GSMA) and the Corrected p-value Meta analysis Method (CPMM). An application to Parkinson's disease is given. Because we had to use secondary data a meta analysis based on original summary values would be desirable.

**Results:**

Data uncertainty by replicated extraction of marker position is shown to be much smaller than 30 cM, a distance up to which a maximum LOD score may usually be found away from the true locus. The main findings are not impaired by data uncertainty.

**Conclusion:**

Applying the proposed method a novel linked region for Parkinson's disease was identified on chromosome 14 (p = 0.036). Comparing the two meta analysis methods we found in this analysis more regions of interest being identified by GSMA, whereas CPMM provides stronger evidence for linkage. For further validation of the extraction method comparisons with raw data would be required.

## Background

Genome wide linkage scans have often been successful in the identification of genes for monogenic diseases. However, the chance of success decreases by the multiplicity of genetic and environmental determinants involved in the aetiology of a complex disease. The contribution of each disease gene to overall risk is presumed to be small, and thus large sample sizes are required to detect the effect [1]. An ad hoc approach is to look for genomic regions that obtain evidence for linkage across several scans, but this provides no direct statistical assessment. A statistically more rigorous and powerful approach to pool results would be a 'mega analysis' using original genotypes and analyze these as a single dataset as suggested by Lander and Kruglyak [2]. Pooling of samples across different studies will increase the sample size and hence help to find loci with small effects. However, one should expect studies to vary in many respects, e.g. ascertainment criteria (multiplex families, sib pair families, and a single large multigenerational family), definition of phenotypes (e.g. diagnostic scheme) and different marker data sets (Marshfield map, Genethon, Decode map). As these marker data sets vary in marker spacing as well as in marker density this leads to further heterogeneity. Moreover, variability in the sample sizes across different studies leads to inconsistencies in the results. Besides that, different ways to incorporate the possible covariates (which are rarely published in detail) are methodological handicaps in a pooled analysis. So, pooling of raw data across several studies needs to be carried out and interpreted with caution. Even though some of these problems cannot be overcome by a meta analysis, pooling of raw data is not necessarily feasible[3].

As the raw genotype data might not always be available to the public, more flexible approaches are required to carry out the meta analysis. In this context Allison and Heo used the Fisher method of combining the p-values across candidate regions in a study of obesity [4]. Province suggested a p-value of 0.72 (= 1/2ln(2)) [5] to overcome the problem of setting all negative evidence against linkage to zero in nonparametric linkage methods. Recently Badner and Gershon [6] proposed an extended approach of combining the p-values across different studies, further on labelled as Corrected p-value Meta analysis Method (CPMM). In CPMM, each reported p-value of a candidate region needs to be transformed by an equation originally given by Lander and Kruglyak [2]. Then, the minimum of these transformed p-values is corrected for the size of the candidate region. Finally Wise etal. [7,8] developed a Genome Search Meta Analysis method (GSMA) specifically to carry out the meta analysis of genome wide linkage searches. GSMA is a nonparametric method based on rank statistics.

If researchers are reluctant to contribute even summary measures like test statistics for linkage (LS), rest assured one may introduce some bias into a meta analysis, similar to publication bias. Additionally, the power of the meta analysis will be decreased. Even if the meta analysis contains an amount of uncertain summary data, the results will provide a higher level of validity than by simply viewing the individual findings. Therefore they are highly valuable in deciding how to proceed next, e.g. which regions to pursue in further studies. That said, one should consider such approaches as preliminary, and the necessity to discuss the impact of data uncertainty onto the findings still remains.

In this study we propose a way to reconstruct test statistics for linkage (LS) and corresponding marker positions as the key summary measure of genome wide scans from condensed materials such as figures in published papers. Furthermore we carry out the meta analysis of all published genome wide scans of susceptibility to PD taking into account the uncertainty of the summary measures by using the GSMA and CPMM.

In this investigation we demonstrate with using PD as an example, how a meta analysis of genome wide linkage searches can be carried out to data with uncertainty when using data extraction. The influence of data uncertainty on the results is discussed and differences between methods are shown.

## Results

By applying inclusion criteria, the meta analysis is based on all published investigations for genome wide linkage to PD as the phenotype of interest: Scottetal. [9], Pankratzetal. [10], DeStefanoetal. [11], Martinezetal. [12] and Hicksetal. [13]. We did not include the genome wide scans by Hampshireetal[14] and Funayamaetal[15], because patients included suffered from the Kufor-Rakebsyndrome or Parkinsonism, respectively.

### Quality of extraction

**Figure 1:**
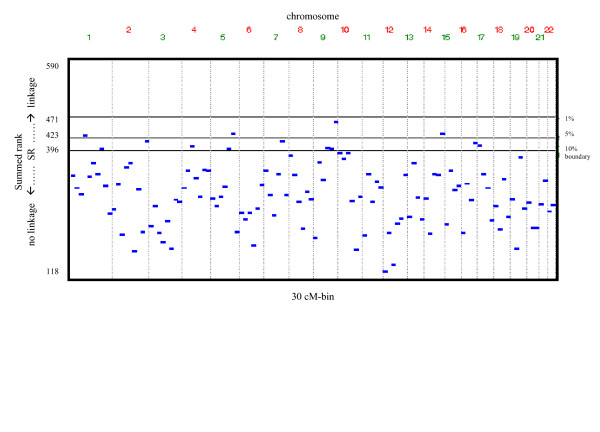
GSMA-results: summed-rank distribution of 30 cM bins. boundary refers to the p-quantil of the distribution of summed ranks assuming no linkage

With the methodology proposed above, we were able to extract the same number of markers (n = 344) as originally investigated by Scottetal. [9]. From the paper by Pankratzetal. [10], the positions and LS of 230 markers could be estimated from figure 2, corresponding to 58% of 400 investigated markers. The corresponding numbers for figure 2 from Hicksetal. [13] are 426 markers (54% of 781 investigated markers), and those for figure 1 from Martinezetal. [12] are 261 markers (67% of 391 investigated markers). DeStefanoetal. [11] provided illustrations for only 4 chromosomes, accounting for the reduced number of extracted markers (12% of 399 investigated markers).

Positions and corresponding LSs of a total of 25 markers were provided in the original papers. The extracted LSs were almost similar to those reported (maximal deviation: 1.15 extracted, 1.24 reported in paper). For two markers the extracted mean positions deviated from those reported by ~12 cM and ~16 cM (corresponding report: table 1,[10]). However, the extracted positions were only 4 cM and 9 cM apart from the sex-averaged locations given by the Marshfield map [16].

**Table 1:** Characteristics of whole genome scans for linkage for Parkinson's disease

Reference	DeStefanoetal. [11]	Scottetal[9]	Hicksetal[13]	Pankratzetal[10]	Martinezetal[12]
Source Population	US	US	Iceland	US	US + Europe
Diagnostic criteria	UKPDS (adapted)	2 cardinal PD-signs + exclusion criteria	2 cardinal PD-signs + Hoen-Yahr crit.	UPDRS (II+III) Diagnosis Check List	3 cardinal PD-signs or 2 signs and at least 30% improvement with levodpoa treatment no parkin mutations
Family Structure	ASP	multiplex families	multiplex families	multiplex families	multiplex families
No. of Families	113	174	51	325 (170ASP)	199 (227 ASP)
No. of Affected	226	378	117	192	471
No. of Marker	392	344	781	400 SNPs	391
Average distance	11 cM	10 cM		8.6 cM	10 cM
Analysis	MLS/NPL	MLOD	Zlr	MLS	MLS
Significant region/markers	9 – D9S1825	5q–D5S816 8p–D8S520 17q – D17S921	1p32–D1S231 D1S2652	2 – D2S206	2p–D2S160 5q–D5S471 6q–D6S257 7p–D7S531 11q–D11S4175 2 – D2S206 19 q – D19S902

*signs compatible with common PD

1 France, United Kingdom, Netherlands, Germany and Italy

2 Weber set 8

3 ABI Prism Linkage Set

ASP	affected sib pair

∅ average

UKPDS UK Parkinson Disease Society Brain Bank Clinical Diagnosis Criteria

UPDRS	Unified Parkinson Disease Rating Scale

MLS Maximum Likelihood Score

MLOD Maximum Parametric LOD Score

NPL Nonparametric Linkage Score

Zlr converted LOD score: v [2ln (10)LOD]

Using our method of extraction, markers yielding higher LS are unambiguously identified in a figure. Thus, we may assume that missed markers are exclusively those with a low value of the LS. Moreover, the estimated standard error of LS ranges from approx. 0 to 0.07 LS-units. The largest deviation was 0.29 LS-units. On average LS of a marker was extracted within a range of 0.06 LS-units. This precision is satisfactory for the coarse grid of genome wide scans.

In order to take into account the uncertainty in marker positions, the estimated standard errors of extracted positions for one marker ranges from approx. 0 to 4.15 cM. The largest span between two single extractions is 28 cM. Position uncertainty was exceptionally high on chromosome X, which was printed with an open ended axis in two figures. Hence, locating markers on this chromosome must be regarded as problematic.

### CPMM

We found evidence for linkage on chromosomes 1 (p = 0.0074) and X (p = 0.0015). In a leave-one-(study-)out cross-validation analysis we did not find any significant linkage. This shows the heterogeneity of the scans, because both findings are primarily caused by the results of one single included genome scan each. The results obtained by CPMM did not reach the level of genome wide significance as suggested by the Lander and Kruglyak criteria [2]. However, they showed a trend towards linkage.

### GSMA

The most significant results by the summed-rank-statistic (SR) could be achieved for the 6^th ^30 cM bin on chromosome 9 (p_SR _= 0.0145). Furthermore, for the 6^th ^30 cM bin on chromosome5 (p_SR _= 0.0363), for the 5^th ^30 cM bin on chromosome 14 (p_SR _= 0.0363) and for the 4^th ^30 cM bin on chromosome 1 (p_SR _= 0.0492) locally significant signals were achieved. The individual ranks for these regions ranged from 51 to 118. Neither for heterogeneity nor for homogeneity between studies evidence was given for any bin (p_het _from 0.1550 to 0.3320). Adjacent to the significant 6^th ^bin, the 4^th ^(p_SR _= 0.0874) and 5^th ^bin (p_SR _= 0.0940) of chromosome 9 showed some trend towards linkage. Similarly, adjacent to the significant 6^th ^bin of chromosome 5, the 5^th ^bin (p_SR _= 0.0940) showed some trend towards linkage. The summed-rank-statistics of each 30 cM bin are shown in figure 1.

The results of the weighted and the unweighted GSMA-analysis were comparable. While the finding of the 6^th ^bin of chromosome 9 got slightly more significant for the weighted-summed rank-statistic (SR_weight_) (p_SR-weight _= 0.0120), the p-value for the 4^th ^bin of chromosome 1 ascended above 5% (p_SR-weight _= 0.0546). In addition, for the 1^st ^bin of chromosome 17 the weighted GSMA-analysis provided a locally significant finding (p_SR _= 0.0752, p_SR-weight _= 0.0460).

The p-values of the findings using 30 cM bins of chromosomes 5 and 9 changed slightly when using 60 cM bins, but they did not fall below 0.00847 (suggestive genome wide evidence). The 3^rd^60 cM bin of chromosome 5 (corresponding to the 5^th ^and 6^th ^30 cM bin) achieved nominal significance by p_SR _= 0.0186 and p_SR-weight _= 0.01438. The 3^rd^60 cM bin of chromosome 9 (corresponding to the 5th and 6^th ^30 cM bin) achieved a nominal significance of p_SR _= 0.0353 and p_SR-weight _= 0.0238.

All these findings remained significant when accounting for data uncertainty by simulation and achieved p-values less than 0.05 in all 333 replications (table 2). No further suggestive evidence for linkage (p < 0.05) can be seen in any of the 333 replications.

**Table 2:** Summary of significant findings of GSMA and CPMM

chromosome	GSMA										CPMM
	30 cM bin	previous reports	location	SR analysis				MC-Replications n (%) from 333*			p-value for whole chromosome
					
				p_SR_	p_SR-weight_	SR*	Ranks*	< 1%	1%–5%	5%–10%	
1	3^th^	Hicks (LOD = 4.9)	58 – 87 cM	0.498	0.661	298	8 – 118	--	--	--	0.007
	4^th^		87 – 116 cM	0.050	0.055	430	51 – 117	--	333 100%	--	
2	4^th^	Martinez (MLS = 2.0)	89 – 120 cM	0.220	0.147	358	39 – 115	--	--	--	0.250
	5^th^		120 – 149 cM	0.183	0.096	368	7 – 118	--	--	8 2%	
	7^th^	Pankratz (LOD = 2.5)	179 – 209 cM	0.447	0.427	308	51 – 73	--	--	--	
5	5^th^	Hicks (LOD = 1.6)	113 – 141 cM	0.094	0.099	399	49 – 125	--	18 5%	315 95%	0.108
	6^th^	Scott (MLOD = 2.4)	142 – 169 cM	0.036	0.034	506	51 – 118	--	333 100%	--	
	3^rd^-60 cM	Martinez (MLS = 1.1)	132 – 198 cM	0.019	0.014	224.5	24.5 – 58	--	304 91%	29 9%	
8	1^st^	Scott (MLOD = 2.0)	0 – 28 cM	0.131	0.208	384.5	14.5 – 117	--	--	--	0.331
9	6^th^	DeStefano (MLS = 1.3)	140 – 169 cM	0.015	0.012	461	55 – 114	--	333 100%	--	0.321
	3^rd^-60 cM		112 – 169 cM	0.035	0.238	215	16 – 58	--	332 99%	1 <1%	
14	5^th^		110 – 138 cM	0.036	0.047	434	51 – 115	--	333 100%		0.300
X		Pankratz (LOD = 2.5)	---- not in GSMA analysis ---								0.002

* for 30 cM bin analysis: SR ranges from 5 to 590, ranks are ranging from 1 to 118

for 60 cM bin analysis: SR ranges from 5 to 290, ranks are ranging from 1 to 58.

** Remaining replications show p ≥ 10%

Chromosomes 2 and 8 are listed, because findings therefore have been reported by individual scans.

## Discussion

We applied data extraction combined with assessing data uncertainty to carry out the meta analysis of genome wide scans of linkage to PD from all published investigations. If known studies without accessible data are not considered, a bias might be introduced in meta analysis so that this problem is reduced by using as much information as possible from published figures. To examine the validity of the extraction method a comparison with all summary measures from the considered genome wide scans would naturally be desirable. Such precise information about LS and the corresponding marker position was reported for only 25 markers in the papers considered. Please note that these markers are those relaying the most outstanding information about linkage. For these markers, we found the precision of the extraction, both for LS and position, to be satisfactory for GSMA where information is pooled within bins of 30 cM size. For the remaining markers, the use of extracted LSs and positions is based on two assumptions: Firstly, missed markers are exclusively those with a low LS. Secondly, a potentially greater uncertainty at markers with lower LS does not have any decisive influence on the results of the meta analysis. This is reasonable, since only the highest LS in each bin is used for GSMA. Furthermore and since none of the bins with exclusively low LS was even suggestively significant in none of the MC-replications, these assumptions may be met. However, a further validation of the extraction method is required. An adequate estimation of sensitivity and specificity of the findings when applying the extraction method can only be achieved by comparing with findings from a pooled analysis of all raw data.

Both meta analysis methods considered here are robust with respect to design, as they can deal with differences in structure and number of families between studies, quantitative and qualitative phenotype definition, genetic markers analysed and methods of statistical analysis. In addition, no assumptions on the mode of inheritance or genetic heterogeneity are necessary for the valid application of these two methods. The distribution and interpretation of the linkage test statistics does depend on the statistical method applied. This is no problem for GSMA, since test statistics are ranked within the single scans. The key information used by CPMM is based on p-values, which may be converted from test statistics for linkage by a known relation. But CPMM requires the raw data to produce reliable results. That was one of the reasons for developing GSMA [17].

To our knowledge no extensive comparison between these methods has been published yet. Thus the relative power of these methods is not yet clear. While with GSMA one searches for evidence for linkage across studies in pre-specified genomic segments (termed as bins), CPMM identifies regions of clustered markers with LS-values indicating towards linkage and assessing significance using p-values corrected for the size of the region. In the presence of uncertainty in marker position it remains unclear which of these approaches remains more powerful or robust. Please note, that it could be problematic to combine the lowest p-values from genome scans particularly for smaller scans, because of a severe bias towards linkage [18]. Giving for instant full weight to very low p-values, CPMM could better detect linkage in the presence of substantial heterogeneity across samples. GSMA might be more powerful when small genetic effects are present in all samples. [17].

Data uncertainty in linkage statistics and marker positions does not deteriorate the strength of the main findings. Since markers are allocated into bins for GSMA, uncertainty in position is reduced to uncertainty of allocation. This allocation is ambiguous only for a small proportion of markers, of which only a small proportion is important for the ranking of bins. Consequently, one might expect less variability in GSMA results due to uncertainty in position. The direct comparison of extracted values of markers to reported values, if available, shows the robustness of the whole approach. The only notable differences appeared from the deviation of original reported marker position to those given by the Marshfield map. In summary, the extraction process led to tolerable uncertainty in both position and test statistic for linkage.

In meta analysis it is important to consider departures from homogeneity between the included studies. For CPMM, the cross-validation as a test of heterogeneity addresses whether the overall results are primarily affected by one single scan. The test of heterogeneous ranks for a bin might lack power when the number of scan is low. So it does not come as a surprise that we were unable to find evidence of either homogeneity or heterogeneity for any of the major findings.

GSMA appears to be robust towards imprecise data extracted from papers reporting genome wide scans. Setting the analysis into a Monte-Carlo framework and comparing results to those of different meta-analytical approaches is a possible way of investigating the sensitivity to uncertainty. However, GSMA and CPMM lead only in parts to concurrent results, applying both methods to our data collection. GSMA came up with more regions of interest, whereas CPMM provided stronger evidence for linkage by lower p-values. Lewis et al. [19] applied the GSMA method to data of families of schizophrenia patients and compared their results with those of a CPMM approach of Badner and Gershon [20]. With GSMA it was possible to identify more significant linkage regions than with CPMM. However, this comparison is limited, because different data sets were included into the meta analyses. Subsequently, there is no evidence to generalise this observation in the comparison of the two methods.

Finally, our approach is limited by the use of uncertain secondary data instead of original summary statistics. Hence, a meta analysis based on all real summary values to verify these preliminary results would be desirable both to further validate our approach and to give further support to the results regarding PD.

### Linkage to PD

GSMA yields weak evidence for linkage to PD for 30 cM bins on chromosomes 1, 5, 9 and 14. While evidence for linkage on chromosome 1 was also provided by CPMM, the findings for chromosomes 5 and 9 remain stable when enlarging the size of the bin to 60 cM or weighting studies according to their number of affected cases included. Additional evidence for linkage was also obtained on chromosome X by CPMM, not detected by GSMA.

We are unable to find a genome wide significant or genome wide suggestive evidence of linkage in our meta analysis based on a total of 1384 affected individuals in 862 families.

The conspicuous 30 cM bin on chromosome 1 (87–116 cM) overlaps with the PARK10 region designated by Hicks et al. [13]. This finding is tally to recently shown genome wide significant associations of SNPs within the PARK10 region [21]. However, in our meta analysis we obtained a linkage signal in this region only if the genome scan from the isolated population in Iceland [13] was included. We could not confirm the evidence of linkage when excluding this most significant single result. Thus, even for the most prominent result we found noticeable heterogeneity among genome scans.

The finding on chromosome 5 (132–198 cM) was yet suspected before [12] by viewing the results of the genome wide scans. Four of these scans found evidence for linkage within a 10 cM interval. Here we attach a p-value of 0.03 (using GSMA) to this result. This is supported by Maraganore et al. [21], who found 2 of eleven associated SNPs (all genome wide significant) located on chromosome 5.

The finding on chromosome 9 (112–169 cM) was highlighted by DeStefanoet al. [11] by a maximum lod score of 1.3 at position 136 cM. This finding is supported by a combination of weaker signals (LS between 0.7 and 1.16) located up to 44 cM apart of two single genome scans [10,12].

The linkage signal on chromosome 14 (110 – 138 cM) arises from the combination of weak signals (LS between 0.62 and 1.6) located within a 9 cM distance of three single genome scans [9,10,13].

Our meta analysis was performed on the basis of only five independent studies. Thus one should regard this finding as an add-in to the list of potential linkage regions.

The findings on chromosomes 9 and 14 supported the results of a whole-genome association study carried out in a sample of idiopathic PD-patients from an isolated population in the Netherlands, recently published by Bertoli-Avella et al[22]. They found strong evidence for association close to the markers D9S1838 (located at 163 cM) and D14S65 (located at 108–129 cM).

## Conclusion

The aetiology of a complex disease like PD is thought to involve several genetic and environmental components and is characterized by a comparatively low genetic heritability. This complicates the search for new candidate genes by genome wide linkage scans. Here, we showed a methodology to extract information from published figures to overcome the bias of inaccessible data. We confirm the evidence of linkage on chromosomes 1, 5 and X. Additionally a signal on chromosome 14 was also obtained which needs confirming replication. With the availability of ultra-high-volume genotyping platforms and 500 K gene chips genome wide association studies should be regarded as a promising addition to already performed linkage scans [21,23,24].

## Methods

### Method of data extraction

Figures presenting test statistics for linkage (LS) were copied from the electronic versions of the original papers into a Microsoft Word^® ^document. We electronically enlarged figures to size A4 in order to gauge crude LS and marker positions on the chromosome, placing arrows from the zero-point to a dot or vertex in the diagram. Length and height values of the arrows were calibrated and rescaled along measurements of the y-axis (linkage statistic) and the chromosome limits plotted along the x-axis (position). More accurate estimates of position could be achieved by placing the arrows at the beginning of each chromosome rather than at the zero-point of the x-axis.

Data extraction was accomplished nine times for each study, each time blinded to previous extractions. In order to take into account the uncertainty in position, extractions were matched, clustering the nearest points. The distance between two points i and j was calculated by dij=f(LSi−LSj)2+(Posi−Posj)2
 MathType@MTEF@5@5@+=feaafiart1ev1aaatCvAUfKttLearuWrP9MDH5MBPbIqV92AaeXatLxBI9gBaebbnrfifHhDYfgasaacH8akY=wiFfYdH8Gipec8Eeeu0xXdbba9frFj0=OqFfea0dXdd9vqai=hGuQ8kuc9pgc9s8qqaq=dirpe0xb9q8qiLsFr0=vr0=vr0dc8meaabaqaciaacaGaaeqabaqabeGadaaakeaacqWGKbazdaWgaaWcbaGaemyAaKMaemOAaOgabeaakiabg2da9maakaaabaGaemOzayMaeiikaGIaemitaWKaem4uam1aaSbaaSqaaiabdMgaPbqabaGccqGHsislcqWGmbatcqWGtbWudaWgaaWcbaGaemOAaOgabeaakiabcMcaPmaaCaaaleqabaGaeGOmaidaaOGaey4kaSIaeiikaGIaemiuaaLaem4Ba8Maem4Cam3aaSbaaSqaaiabdMgaPbqabaGccqGHsislcqWGqbaucqWGVbWBcqWGZbWCdaWgaaWcbaGaemOAaOgabeaakiabcMcaPmaaCaaaleqabaGaeGOmaidaaaqabaaaaa@4EA4@, with f as a factor to correct for different scales (LS-units vs. cM). It also can be used to give higher weights to differences of LS than to that of positions, since neighbouring points can be distinguished more easily by LS than by position. A value of f = 8 was empirically found useful showing no clear mismatch.

The quality of data extraction was separately checked by visual inspection for each extraction and for the mean of extractions. Finally, the mean and the standard error of matched extracted LS and positions were calculated and used for the subsequent meta analysis. Standard errors for markers extracted only once were defined as equal to the median standard error of all remaining markers. We directly used LS when LS and corresponding marker positions were reported in the articles. In this case standard errors were set to zero.

### Methods of meta analysis

#### CPMM

CPMM is based on p-values for linkage peaks. Badner and Gershon [6] suggested that those nominal p-values per locus have to be corrected for genome wide testing, because evidence for linkage can occur in a region of up to 30 cM away from the disease susceptible locus[6]. They refer to Feingold et al. [25], who estimated the probability p* for the minimum p-value being observed within such a linkage region.

This corrected p-value for such a region is, p∗=1−(1−p)C+2λG⋅Z(p)⋅φ[Z(p)]⋅ν[Z(p)4λΔ]
 MathType@MTEF@5@5@+=feaafiart1ev1aaatCvAUfKttLearuWrP9MDH5MBPbIqV92AaeXatLxBI9gBaebbnrfifHhDYfgasaacH8akY=wiFfYdH8Gipec8Eeeu0xXdbba9frFj0=OqFfea0dXdd9vqai=hGuQ8kuc9pgc9s8qqaq=dirpe0xb9q8qiLsFr0=vr0=vr0dc8meaabaqaciaacaGaaeqabaqabeGadaaakeaacqWGWbaCdaahaaWcbeqaaiabgEHiQaaakiabg2da9iabigdaXiabgkHiTiabcIcaOiabigdaXiabgkHiTiabdchaWjabcMcaPmaaCaaaleqabaGaem4qameaaOGaey4kaSIaeGOmaidcciGae83UdWMaem4raCKaeyyXICTaemOwaOLaeiikaGIaemiCaaNaeiykaKIaeyyXICTae8NXdyMaei4waSLaemOwaOLaeiikaGIaemiCaaNaeiykaKIaeiyxa0LaeyyXICTae8xVd4Maei4waSLaemOwaOLaeiikaGIaemiCaaNaeiykaKYaaOaaaeaacqaI0aancqWF7oaBcqqHuoaraSqabaGccqGGDbqxaaa@5D7F@ where the notation is as follows: p denotes the Bonferroni corrected point-wise p-value from each scan to take multiple testing into account. C denotes the number of chromosomes. λ denotes the rate of crossovers per Morgan given by Lander and Kruglyak [2]; it depends on the analysis method and family structure. G denotes the size of the linkage region in Morgan, here G = 60 cM. Z (p) denotes the standard normal inverse of p. φ[Z(*p*)] the density function of the normal distribution. Δ denotes the average marker spacing in Morgan. ν (x) denotes the discreteness correction for the distance between markers; for x <2 we have v (x) ≈ exp (-0.583x).

This equation differs from that used by Badner and Gershon[6] and given by Feingoldetal[25]. The first term pC was replaced by 1- (1-p)^C ^because observed p-values less significant than 0.045 (LOD-scores of ~0.89) result in p* > 1. Applying CPMM, we proceeded as follows: On each chromosome the most significant marker, defined by the maximum LS, was identified across all scans. A region ± 30 cM around this marker was considered a linkage region if p* < 0.01. Hence, all LS of the remaining scans within a linkage region were converted to p-values by using Holman's triangle[26] as implemented in the Nyholt table[27]. For the X chromosome we followed the X-linked MLS approach as suggested by Cordell et al[28]. These p-values were further corrected yielding the corresponding p*-values as described in the above equation. The p-values of markers outside the linkage region were set to 0.72 (= 1/2ln(2)) as suggested by Province [5].

For each region the multiple scan probability MSP:p=p(χ1−α,df=12>Y2)
 MathType@MTEF@5@5@+=feaafiart1ev1aaatCvAUfKttLearuWrP9MDH5MBPbIqV92AaeXatLxBI9gBaebbnrfifHhDYfgasaacH8akY=wiFfYdH8Gipec8Eeeu0xXdbba9frFj0=OqFfea0dXdd9vqai=hGuQ8kuc9pgc9s8qqaq=dirpe0xb9q8qiLsFr0=vr0=vr0dc8meaabaqaciaacaGaaeqabaqabeGadaaakeaacqWGnbqtcqWGtbWucqWGqbaucqGG6aGocqWGWbaCcqGH9aqpcqWGWbaCcqGGOaakiiGacqWFhpWydaqhaaWcbaGaeGymaeJaeyOeI0Iae8xSdeMaeiilaWIaemizaqMaemOzayMaeyypa0JaeGymaedabaGaeGOmaidaaOGaeyOpa4JaemywaK1aaWbaaSqabeaacqaIYaGmaaGccqGGPaqkaaa@45F3@ was calculated with Y2=∑−2log⁡(p∗⋅i) for i=1 to n
 MathType@MTEF@5@5@+=feaafiart1ev1aaatCvAUfKttLearuWrP9MDH5MBPbIqV92AaeXatLxBI9gBaebbnrfifHhDYfgasaacH8akY=wiFfYdH8Gipec8Eeeu0xXdbba9frFj0=OqFfea0dXdd9vqai=hGuQ8kuc9pgc9s8qqaq=dirpe0xb9q8qiLsFr0=vr0=vr0dc8meaabaqaciaacaGaaeqabaqabeGadaaakeaacqWGzbqwdaahaaWcbeqaaiabikdaYaaakiabg2da9maaqaeabaGaeyOeI0IaeGOmaiJagiiBaWMaei4Ba8Maei4zaCMaeiikaGIaemiCaa3aaWbaaSqabeaacqGHxiIkaaGccqGHflY1cqWGPbqAcqGGPaqkcqqGGaaicqWGMbGzcqWGVbWBcqWGYbGCcqqGGaaicqWGPbqAcqGH9aqpcqaIXaqmcqqGGaaicqWG0baDcqWGVbWBcqqGGaaicqWGUbGBaSqabeqaniabggHiLdaaaa@4EE0@. n denotes the number of scans considered, as originally suggested by RA. Fisher in 1932[6].

According to the criteria for genome scans by Lander and Kruglyak[2] we considered a linkage signal as suggestive following application of CPMM when p = 0.0007 and as significant when p = 0.00002. Cross-validation analysis excluding the most significant result was carried out if CPMM analysis yielded p ≤ 0.001[6].

#### GSMA

Briefly, the GSMA [7,8] method assesses evidence for linkage by splitting all chromosomes into N bins of approximately equal size. For each genome scan included, the most significant LS is recorded. Bins are then ranked in order of significance with the most significant bin assigned rank N. Equal test statistics for several bins within a study were assigned tied ranks. The ranks of bins are summed across the genome scans. This summed-rank-statistic (SR) is compared to the critical values of a summed-rank-distribution (Edgeworth series approximation[29]) under the null hypothesis of no linkage. We also carried out a weighted GSMA analysis. For this each rank was multiplied by its study weight (N(affected cases)
 MathType@MTEF@5@5@+=feaafiart1ev1aaatCvAUfKttLearuWrP9MDH5MBPbIqV92AaeXatLxBI9gBaebbnrfifHhDYfgasaacH8akY=wiFfYdH8Gipec8Eeeu0xXdbba9frFj0=OqFfea0dXdd9vqai=hGuQ8kuc9pgc9s8qqaq=dirpe0xb9q8qiLsFr0=vr0=vr0dc8meaabaqaciaacaGaaeqabaqabeGadaaakeaadaGcaaqaaiabd6eaojabcIcaOiabdggaHjabdAgaMjabdAgaMjabdwgaLjabdogaJjabdsha0jabdwgaLjabdsgaKjabbccaGiabdogaJjabdggaHjabdohaZjabdwgaLjabdohaZjabcMcaPaWcbeaaaaa@41DC@, divided by the mean of this value of all studies) before summed up to another summed-rank-statistic SR_weight _[17].

For the analysis we did not consider the X chromosome. The X chromosome was drawn on an open end scale in some of the figures. Hence the position of the extracted markers could only be determined rather imprecisely[14,15].

We considered an approximate bin size of 30 cM as recommended by Wiseetal[7]. In total 118 bins were used. SR across all 5 studies ranged from 5 to 590.

For each bin we calculated p-values of three kinds of tests. First, p_SR _gives the probability of an arbitrary bin to achieve the observed SR or a higher value. SR analysis assesses the significance of each bin independently. Applying Bonferroni correction for the number of bins, significant genome wide evidence for linkage of 5%, as defined by Lander and Kruglyak [2], will be equivalent to p_SR_<0.00042 for 118 30 cM bins (expected once by chance in 20 meta analyses). Suggestive evidence (expected once by chance per single meta analysis) is given for a p_SR _< 0.00847.

Secondly, p_het _gives the probability of heterogeneous ranks across studies for a bin, conditional on the observed rank sum. Therefore we used Qj=∑(Rij−R¯⋅j)2
 MathType@MTEF@5@5@+=feaafiart1ev1aaatCvAUfKttLearuWrP9MDH5MBPbIqV92AaeXatLxBI9gBaebbnrfifHhDYfgasaacH8akY=wiFfYdH8Gipec8Eeeu0xXdbba9frFj0=OqFfea0dXdd9vqai=hGuQ8kuc9pgc9s8qqaq=dirpe0xb9q8qiLsFr0=vr0=vr0dc8meaabaqaciaacaGaaeqabaqabeGadaaakeaacqWGrbqudaWgaaWcbaGaemOAaOgabeaakiabg2da9maaqaeabaGaeiikaGIaemOuai1aaSbaaSqaaiabdMgaPjabdQgaQbqabaGccqGHsislcuWGsbGugaqeamaaBaaaleaacqGHflY1cqWGQbGAaeqaaOGaeiykaKYaaWbaaSqabeaacqaIYaGmaaaabeqab0GaeyyeIuoaaaa@3F74@ as test statistic, proposed by Zintzaras and Ioannidis[30,31], where R_ij _is the rank of j-th bin in the i-th study and R¯ij
 MathType@MTEF@5@5@+=feaafiart1ev1aaatCvAUfKttLearuWrP9MDH5MBPbIqV92AaeXatLxBI9gBaebbnrfifHhDYfgasaacH8akY=wiFfYdH8Gipec8Eeeu0xXdbba9frFj0=OqFfea0dXdd9vqai=hGuQ8kuc9pgc9s8qqaq=dirpe0xb9q8qiLsFr0=vr0=vr0dc8meaabaqaciaacaGaaeqabaqabeGadaaakeaacuqGsbGugaqeamaaBaaaleaacqqGPbqAcqqGQbGAaeqaaaaa@30CF@ is the mean rank of the j-th bin across studies. A small p_het _indicates consistent evidence for linkage across studies, while a large p_het _indicates heterogeneity between the considered searches.

We assigned top ranks to known bins and the mean of the remaining ranks to empty bins[7] to overcome the problem of missing values.

#### Sensitivity analysis

A Monte Carlo (MC) simulation approach[32] was used to determine the change in SR due to data uncertainty for LS and position caused by the extraction process. The simulations were replicated 333 times (replication number limited by computer time) while randomly drawing a marker position and LS from normal distributions, using mean and standard error from data extraction.

The original studies forming the basis of this meta analysis were all carried out in accordance with the Declaration of Helsinki.

### Literature selection of genome wide scans for Parkinson's disease

We carried out a literature search in MEDLINE for MESH-headings Genetics, Parkinson's disease and genome scan (or screening), restricted from 1998 to 2004 and sourced references of neurological and genetic journals. In total we were able to identify seven genome wide linkage scans of Parkinson's disease [9-15]. Three family samples have been reanalysed and published twice. Recently a genome wide association of PD study was published, that we used only for comparing results [21].

#### Inclusion/exclusion criteria for genome wide scan

The following criteria for the inclusion of genome wide scans in the meta analysis were defined to ensure the quality of the individual studies and the data to be extracted:

1. Patients are included by status of Parkinson's disease and not being selected e.g. by family history or therapy response.

2. Statistical results are available in figures or tables for whole chromosomes, at least for the major findings.

3. The statistical analysis is carried out by using established genetic epidemiological methods.

4. The analysis concentrates exclusively on the susceptibility to PD, not e.g. to the age of onset. Thus the two genome scans based on other phenotypes are excluded [7].

The study characteristics of the five identified and included genome wide scans on susceptibility to PD are given in table 1.

## Abbreviations

GSMA Genome Search Meta Analysis method

CPMM Corrected p-value Meta analysis Method

LS linkage statistic

SR summed-rank statistic

PD Parkinson's disease

## Authors' contributions

AR participated in the design of the project, carried out the data extraction and performed the meta analysis.

MS participated in the design of the project and carried out the performed the meta analysis.

BMM, ThG and HB participated in the design of the project and helped to draft the manuscript.

All authors read and approved the final manuscript.

## References

[B1] Risch N, Merikangas K (1996). The future of genetic studies of complex human diseases. Science.

[B2] Lander E, Kruglyak L (1995). Genetic dissection of complex traits: guidelines for interpreting and reporting linkage results. Nat Genet.

[B3] Bravata DM, Olkin I (2001). Simple pooling versus combining in meta-analysis. Eval Health Prof.

[B4] Allison DB, Heo M (1998). Meta-analysis of linkage data under worst-case conditions: a demonstration using the human OB region. Genetics.

[B5] Province MA (2001). The significance of not finding a gene. Am J Hum Genet.

[B6] Badner JA, Gershon ES (2002). Regional meta-analysis of published data supports linkage of autism with markers on chromosome 7. Mol Psychiatry.

[B7] Wise LH, Lanchbury JS, Lewis CM (1999). Meta-analysis of genome searches. Ann Hum Genet.

[B8] Wise LH, Lewis CM (1999). A method for meta-analysis of genome searches: application to simulated data. Genet Epidemiol.

[B9] Scott WK, Nance MA, Watts RL, Hubble JP, Koller WC, Lyons K, Pahwa R, Stern MB, Colcher A, Hiner BC, Jankovic J, Ondo WG, Allen FH, Goetz CG, Small GW, Masterman D, Mastaglia F, Laing NG, Stajich JM, Slotterbeck B, Booze MW, Ribble RC, Rampersaud E, West SG, Gibson RA, Middleton LT, Roses AD, Haines JL, Scott BL, Vance JM, Pericak-Vance MA (2001). Complete genomic screen in Parkinson disease: evidence for multiple genes. JAMA.

[B10] Pankratz N, Nichols WC, Uniacke SK, Halter C, Rudolph A, Shults C, Conneally PM, Foroud T (2002). Genome screen to identify susceptibility genes for Parkinson disease in a sample without parkin mutations. Am J Hum Genet.

[B11] DeStefano AL, Golbe LI, Mark MH, Lazzarini AM, Maher NE, Saint-Hilaire M, Feldman RG, Guttman M, Watts RL, Suchowersky O, Lafontaine AL, Labelle N, Lew MF, Waters CH, Growdon JH, Singer C, Currie LJ, Wooten GF, Vieregge P, Pramstaller PP, Klein C, Hubble JP, Stacy M, Montgomery E, MacDonald ME, Gusella JF, Myers RH (2001). Genome-wide scan for Parkinson's disease: the GenePD Study. Neurology.

[B12] Martinez M, Brice A, Vaughan JR, Zimprich A, Breteler MM, Meco G, Filla A, Farrer MJ, Betard C, Hardy J, De Michele G, Bonifati V, Oostra B, Gasser T, Wood NW, Durr A (2004). Genome-wide scan linkage analysis for Parkinson's disease: the European genetic study of Parkinson's disease. J Med Genet.

[B13] Hicks AA, Petursson H, Jonsson T, Stefansson H, Johannsdottir HS, Sainz J, Frigge ML, Kong A, Gulcher JR, Stefansson K, Sveinbjornsdottir S (2002). A susceptibility gene for late-onset idiopathic Parkinson's disease. Ann Neurol.

[B14] Hampshire DJ, Roberts E, Crow Y, Bond J, Mubaidin A, Wriekat AL, Al Din A, Woods CG (2001). Kufor-Rakeb syndrome, pallido-pyramidal degeneration with supranuclear upgaze paresis and dementia, maps to 1p36. J Med Genet.

[B15] Funayama M, Hasegawa K, Kowa H, Saito M, Tsuji S, Obata F (2002). A new locus for Parkinson's disease (PARK8) maps to chromosome 12p11.2-q13.1. Ann Neurol.

[B16] Broman KW, Murray JC, Sheffield VC, White RL, Weber JL (1998). Comprehensive human genetic maps: individual and sex-specific variation in recombination. Am J Hum Genet.

[B17] Levinson DF, Levinson MD, Segurado R, Lewis CM (2003). Genome scan meta-analysis of schizophrenia and bipolar disorder, part I: Methods and power analysis. Am J Hum Genet.

[B18] Goring HH, Terwilliger JD, Blangero J (2001). Large upward bias in estimation of locus-specific effects from genomewide scans. Am J Hum Genet.

[B19] Lewis CM, Levinson DF, Wise LH, DeLisi LE, Straub RE, Hovatta I, Williams NM, Schwab SG, Pulver AE, Faraone SV, Brzustowicz LM, Kaufmann CA, Garver DL, Gurling HM, Lindholm E, Coon H, Moises HW, Byerley W, Shaw SH, Mesen A, Sherrington R, O'Neill FA, Walsh D, Kendler KS, Ekelund J, Paunio T, Lonnqvist J, Peltonen L, O'Donovan MC, Owen MJ, Wildenauer DB, Maier W, Nestadt G, Blouin JL, Antonarakis SE, Mowry BJ, Silverman JM, Crowe RR, Cloninger CR, Tsuang MT, Malaspina D, Harkavy-Friedman JM, Svrakic DM, Bassett AS, Holcomb J, Kalsi G, McQuillin A, Brynjolfson J, Sigmundsson T, Petursson H, Jazin E, Zoega T, Helgason T (2003). Genome scan meta-analysis of schizophrenia and bipolar disorder, part II: Schizophrenia. Am J Hum Genet.

[B20] Badner JA, Gershon ES (2002). Meta-analysis of whole-genome linkage scans of bipolar disorder and schizophrenia. Mol Psychiatry.

[B21] Maraganore DM, de Andrade M, Lesnick TG, Strain KJ, Farrer MJ, Rocca WA, Pant PVK, Frazer KA, Cox DR, Ballinger DG (2005). High-Resolution Whole-Genome Association Study of Parkinson Disease. Am J Hum Genet.

[B22] Bertoli-Avella AM, Dekker MC, Aulchenko YS, Houwing-Duistermaat JJ, Simons E, Testers L, Pardo LM, Rademaker TA, Snijders PJ, van Swieten JC, Bonifati V, Heutink P, van Duijn CM, Oostra BA (2006). Evidence for novel loci for late-onset Parkinson's disease in a genetic isolate from the Netherlands. Hum Genet.

[B23] Tu IP, Whittemore AS (1999). Power of association and linkage tests when the disease alleles are unobserved. Am J Hum Genet.

[B24] Thomas DC, Haile RW, Duggan D (2005). Recent developments in genomewide association scans: a workshop summary and review. Am J Hum Genet.

[B25] Feingold E, Brown PO, Siegmund D (1993). Gaussian models for genetic linkage analysis using complete high-resolution maps of identity by descent. Am J Hum Genet.

[B26] Holmans P (1993). Asymptotic properties of affected-sib-pair linkage analysis. Am J Hum Genet.

[B27] Nyholt DR (2000). All LODs are not created equal. Am J Hum Genet.

[B28] Cordell HJ, Kawaguchi Y, Todd JA, Farrall M (1995). An extension of the Maximum Lod Score method to X-linked loci. Ann Hum Genet.

[B29] Koziol JA, Feng AC (2004). A note on the genome scan meta-analysis statistic. Ann Hum Genet.

[B30] Zintzaras E, Ioannidis JP (2005). Heterogeneity testing in meta-analysis of genome searches. Genet Epidemiol.

[B31] Zintzaras E, Ioannidis JP (2005). HEGESMA: genome search meta-analysis and heterogeneity testing. Bioinformatics.

[B32] Morgan MG, Henrion M, Small M (1990). Uncertainty
a guide to dealing with uncertainty in quantitative risk and policy analysis.

